# Food Insecurity, Poor Diet Quality, and Suboptimal Intakes of Folate and Iron Are Independently Associated with Perceived Mental Health in Canadian Adults

**DOI:** 10.3390/nu9030274

**Published:** 2017-03-14

**Authors:** Karen M. Davison, Lovedeep Gondara, Bonnie J. Kaplan

**Affiliations:** 1 School of Nursing, University of British Columbia and Health Science Program, Department of Biology, Kwantlen Polytechnic University, Surrey, BC V3W 2M8, Canada; 2 Department of Computer Science, University of Illinois Springfield and Department of Computer Science, Simon Fraser University, Burnaby, BC V5A 1S6, Canada; lvdp00@gmail.com; 3 Cumming School of Medicine, University of Calgary, Calgary, AB T2N 1N4, Canada; kaplan@ucalgary.ca

**Keywords:** food insecurity, diet quality, nutrient intakes, mental health

## Abstract

Background: To address nutrition-related population mental health data gaps, we examined relationships among food insecurity, diet quality, and perceived mental health. Methods: Stratified and logistic regression analyses of respondents aged 19–70 years from the Canadian Community Health Survey, Cycle 2.2 were conducted (*n* = 15,546). Measures included the Household Food Security Survey Module, diet quality (i.e., comparisons to the *Dietary Reference Intakes*, Healthy Eating Index), perceived mental health (poor versus good), sociodemographics, and smoking. Results: In this sample, 6.9% were food insecure and 4.5% reported poor mental health. Stratified analysis of food security and mental health status by age/gender found associations for poor diet quality, protein, fat, fibre, and several micronutrients (*p*-values < 0.05); those who were food insecure tended to have higher suboptimal intakes (*p*-values < 0.05). After adjustment for covariates, associations in relation to mental health emerged for food insecurity (OR = 1.60, 95% CI 1.45–1.71), poor diet quality (1.61, 95% CI 1.34–1.81), and suboptimal intakes of folate (OR = 1.58, 95% CI 1.17–1.90) and iron (OR = 1.45, 95% CI 1.23–1.88). Conclusions: Population approaches that improve food security and intakes of high quality diets may protect people from poor mental health.

## 1. Introduction

A growing body of evidence indicates relationships among food insecurity, diet quality, and mental health; however, their simultaneous effects are rarely studied. Food insecurity occurs when people are physically or economically unable to consume a sufficient quantity of food or have uncertainty in their ability to do so [1], and it has been associated with various indicators of mental ill health such as depression, mania, disordered eating, impaired cognition, higher internalizing and externalizing behaviours, and suicidal ideation [2,3,4,5]. For indicators such as depression, the links with food insecurity and nutrition may be bidirectional [6]. Diet quality, which encompasses adequacy or sufficiency, moderation, variety or diversity, and balance or equilibrium of nutrient and food intakes [7], is also critical to mental health. The two concepts of food insecurity and diet quality are interrelated: food insecurity includes the components of insufficient food quantity and quality, feelings of deprivation, and disrupted eating [8], and so it is inversely associated with higher levels of diet quality [9].

The understanding about the relationship between food insecurity and diet quality with respect to mental health is evolving. Food insecurity can contribute to over- and under-nutrition, nutrient excesses, disproportions, and deficiencies, as well as eating disturbances [10,11,12]. Manifestations of nutritional deficiencies include psychiatric symptoms, and single nutrients such as omega-3 fatty acids and folate have received attention in epidemiologic and intervention studies targeting mental health [13,14]. Intervention studies that have utilized multi-nutrient formulas with both minerals and vitamins have shown an even greater benefit for mental health [15], indicating the need for all essential nutrients for optimal mental function. Studies of adults with mood disorders, for example, have indicated that when compared to a general population, a larger proportion had suboptimal intakes of essential nutrients [16]. Furthermore, nutrient intakes have been found to be correlated with psychological functioning [17], and when food insecurity was also present, nutrient intakes and mania symptoms were worse [18]. Eating disturbances (e.g., fasting, binging), which can impact nutritional status, are also associated with both food insecurity and mental health [19,20,21]. Compromised diet quality caused by food insecurity may also contribute to alterations in the gut–microbiota–brain axis [22], creating metabolic, immune, and inflammatory responses [23] that contribute to worsening mental health. Conversely, intake of a good quality diet combined with food security can contribute to anti-inflammatory and protective effects of various nutrients and other bioactive components that optimize the brain’s biochemistry and support cognitive health.

While both food security and diet quality are critical to mental health, there has been limited research examining their relationships. Specifically, there are knowledge gaps about how food insecurity may impact nutrient intakes in the context of poor and good levels of mental health and how these relationships may be influenced by different determinants of health such as income. Furthermore, despite burgeoning research in this area, studies have focused on specific mental health conditions and have narrowly defined a “good outcome” as a reduction in symptoms of mental illness and/or improved functional ability. In recent years, however, the concept of mental health has moved to a broader interest in individuals’ appraisals about their experiences and the meaning that they attribute to these experiences [24], as this may impact health service use.

There has been increasing recognition that barriers to better nutrition must be understood at the population level among groups who are most likely to have poorer-quality diets and to be at risk for mental health conditions. Therefore, examining measures of mental health in a general population and their potential associations with food insecurity and diet quality can provide direction on population-level interventions and policies that foster mental health for individuals without diagnosed mental health conditions, potentially prevent or delay the development of a mental health condition, and optimize outcomes for those with mental health conditions that are symptomatic. The importance of this research is emphasized by the fact that food security status and diet quality are modifiable. To help address these knowledge gaps, we analysed data from a large well-designed cross-sectional study to examine: (1) differences in energy and nutrient intakes by food security and perceived mental health status; (2) differences in adequacy of macro- and micronutrient intakes by food security and perceived mental health status; and (3) the simultaneous effects of food insecurity, diet quality, and suboptimal micronutrient intakes in relation to perceived mental health. We hypothesized that: (1) diet quality is compromised by poor mental health and food insecurity; and (2) food insecurity and diet quality are independent predictors of poor mental health.

## 2. Materials and Methods

### 2.1. Sample

The sample was derived from cycle 2.2 (Nutrition) of the Canadian Community Health Survey (2004) conducted by Statistics Canada [25]. A complete description of the survey data is provided in documentation from Health Canada [26]. The survey sample consisted of 35,107 respondents (0 years+) living in private residences in Canada’s 10 provinces. Initial interviews were conducted in-person and collected information about the respondent’s demographics, general health, dietary intake (24-h recall), and food security. A second 24-h recall was conducted by telephone with 10,786 respondents 3 to 10 days after the initial interview. For our analysis, we used data from adult respondents aged 19 to 70 (*n* = 20,498). Adults were selected, as associations between food insecurity and dietary quality is less consistently associated with low dietary quality in children. This is believed to be due to adults shielding children from compromised diets in the context of household food insecurity [9]. Thus, those less than 19 years of age (*n* = 15,190) were excluded. In addition, adults aged 70 years+ (*n* = 4371) were excluded, as there was limited data available on the second 24-h diet recalls in this age group.

### 2.2. Measures

#### 2.2.1. Perceived Mental Health

The variable “perceived mental health” is a general indicator of individuals who are suffering from some form of mental disorder, mental or emotional problems, or distress, which is not necessarily reflected in the more global measure “self-perceived health” [27], and can affect service use [27,28]. This measure of overall mental health status is considered to align with the World Health Organization’s definition of mental health where a person with or without a diagnosed mental health condition can experience well-being in which they realize their own abilities, can cope with the normal stresses of life, can work productively, and contribute to their community [29]. Research suggests perceived mental health is associated with mental morbidity measures such as non-specific psychological distress, depressive symptoms, activity limitations, and physical and emotional role functioning [30]. Strong positive associations between all mental morbidity measures and perceived mental health have been reported, with stronger associations between past month prevalence as compared to past 2- to 12-month prevalence and lifetime disorder [30].

Respondents rated perceived mental health based on answers to the question: “How would you say your mental health is? Excellent? Very good? Good? Fair? Poor?” The responses were dichotomized as poor/fair (poor mental health) and good/very good/excellent (good mental health). This treatment of the perceived mental health measure has been done in other studies [31,32], and is an established approach to modifying self-reported health measures [33].

#### 2.2.2. Food Security

Food security was measured using the 18-item Household Food Security Survey Module (HFSSM) [34]. Food insecurity was determined based on affirmative responses to either of the “food sufficiency questions” which asked whether the household, in the past 12 months, sometimes did not have enough to eat or often did not have enough to eat. Food security status was classified as: (1) Food secure: access at all times in the previous year to enough food for an active, healthy life for all household members; (2) Food insecure: included categories of moderate or severe food insecurity where any household member had compromises in quality and/or quantity of food consumed which may have disrupted eating patterns.

#### 2.2.3. Diet Quality

There are various diet quality indicators, and it is recommended that more than one be selected when testing associations with health outcomes [35]. For the current study, the indicators of diet quality included measures of energy and nutrient intakes and comparisons to North American dietary standards, as well as an index of diet quality. Nutrient intakes were compared to the Dietary Reference Intakes (DRIs) [36] by specified age/gender categories to review patterns of nutrient inadequacies. To assess intakes of the major nutrients, the Acceptable Macronutrient Distribution Ranges (AMDR) were used as the reference. The AMDR is a range of intakes for a particular energy source that is expressed as a percentage of total energy intakes, and is associated with reduced risk of chronic disease while providing adequate intakes of essential nutrients [37]. To estimate the prevalence of potentially inadequate micronutrient nutrient intakes, the Estimated Average Requirement (EAR) cut point method of the *Dietary Reference Intakes* was used. The EAR is a nutrient intake value that is estimated to meet the requirements of half of the healthy individuals in a group. For nutrients with an EAR, the percentage below the EAR reflects the prevalence of potentially inadequate intakes for a given nutrient. For iron, “the full probability approach” [36] was used, which accounts for skewness in the requirement distributions of this nutrient.

The Canadian Healthy Eating Index (HEI) [35] was also used as an indicator of diet quality. The HEI includes nine scored components of the intakes of the four food groups of the Canadian Food Guide [38], total fat (percent of energy intake), saturated fat (percent of energy intake), total cholesterol intake, total sodium intake, and diet variety. The diet variety component is based on having at least one serving from each food group (i.e., vegetables and fruit, grain products, meat and alternatives, milk and alternatives). A final score of ≤50 indicates poor diet quality, 51 to 80 indicates a diet that needs improvements, and 81 to 100 indicates good diet quality.

#### 2.2.4. Covariates

The covariates sex, age (categories of 19–30, 31–50, 51–70 years), income (five categories: lowest income, lower middle income, middle income, upper middle income, highest income), education (secondary school graduate or less vs. education above secondary school level), relationship status (married or common-law vs. single), and smoking status (current daily smoker vs. not a current daily smoker) were also included, as these factors can influence dietary intakes and food security. A five-level variable describes income adequacy according to total household income in the past 12 months and the number of people in the household. The highest level of education obtained by the respondent was classified as less than secondary school graduation, secondary school graduation, some postsecondary education, and postsecondary graduation. Relationship status included the categories of married, common-law, widowed/separated/divorced, and single/never married. A dichotomous variable differentiated those who were and were not current daily smokers (i.e., smoking daily or occasionally at the present time).

### 2.3. Analysis

The secured data was analysed in the Statistics Canada Research Data Centre at the University of British Columbia using SAS (version 9.1, 2003, SAS Institute, Cary, NC, USA) and SIDE-IML (Software for Intake Distribution Estimation in IML language, version 1.11, 2001, Iowa State University, Ames, IA, USA). Survey weights are incorporated into the calculations to provide national representation [25]. To account for survey design effects, the bootstrap resampling technique was used [39].

To assess for quality in dietary intake reporting, energy intake (EI) was examined in relation to estimated energy requirements (EER) [40,41] based on respondents’ sex, age, self-reported physical activity level, as well as self-reported or measured height and weight to estimate requirements [42]. The physical activity coefficients used in the EER equation were based on three levels which account for the frequency and duration of activity: active, moderately active, or inactive [26].

To compare differences between food security status and nutrient intakes in relation to mental health, Mann–Whitney *U* tests, chi-square tests, ANOVAs, or their non-parametric equivalents (e.g., Kruskal–Wallis tests) were used where applicable. The nutritional adequacy of intakes by age/sex group and food security status was determined using data from the first (full sample) and second (subsample) dietary recalls [25]. The prevalence of potentially inadequate intakes were estimated using the EAR cut point approach for nutrients which have an EAR, accounting for age/sex categories and increased requirements of vitamin C in current smokers [43,44,45,46,47].

To examine relationships between the outcome variable mental health (poor vs. good) in relation to food security, diet quality, and suboptimal intakes of micronutrient intakes as defined by EAR cut-offs, logistic regression analysis was conducted which controlled for the covariates. Goodness-of-fit chi-squared tests were applied to assess for model fit. The level of significance for all statistical tests was *p* < 0.05.

## 3. Results

### 3.1. Sample

The sample consisted of more females (55%) than males (45%); about two-thirds (68.4%) of the sample were less than 50 years. Almost 7% (6.9%) were food insecure, and 4.5% reported poor or fair mental health. Within the adult sample (19 to 70 years), about 8% (7.8%) were post-secondary graduates, 44.1% earned an income considered to be at the lower middle range or less, and 50.1% were in a relationship.

### 3.2. Bivariate Analyses

The medians of EI:EER tended to be lower among those who reported food insecurity, however no significant differences were found (Table S1). Poor quality diets as defined by the HEI were more prevalent among those with poor mental health (33.6%) compared to those with good mental health (26.9%; *p* < 0.001), and there were significant associations between food security status, diet quality, and mental health (all *p*-values < 0.05). For macronutrient and fibre intakes, there were significant associations between mental health and food insecurity for protein (grams) in males between 31 to 50 years and 51 to 70 years, carbohydrates (g) and fat (g) in females between 31 to 50 years and 51 to 70 years, and fibre (g) in males between 31 to 50 years and females 51 to 70 years (Table S1). For micronutrients, there was a relatively consistent trend observed for intakes by gender categories where those with good mental health and food secure status had higher intakes than those reporting poor mental health and food insecure status (Table S2). For females between 19 to 30 years, vitamin C intakes were significantly lower for those with poor mental health and food insecure status (*p* < 0.05). Thiamin and folate intakes among males and females 31 to 50 years were also significantly lower for those with poor mental health and food insecure status. For males in the same age group, significantly lower intakes of vitamins B_3_, B_6_, and B_9_, as well as the minerals phosphorus, potassium, and zinc in those with poor mental health and food insecure status. For males and females 51 to 70 years, there was significant association for vitamin C when stratified by mental health and food security. For females in this age range, many significant associations were also found that included vitamins A, B_1_, B_2_, B_3_, B_6_, B_9_, B_12_, and D, as well as the minerals magnesium, phosphorus, and potassium. Interestingly, for many of the B vitamins, intakes were slightly higher among females with food insecure status who reported poor mental health.

When compared to the DRIs, a significantly higher proportion of respondents that were food insecure had protein intakes below the AMDRs, regardless of mental health status (Table 1). A significantly higher proportion of the sample with food insecure and poor mental health status had micronutrient intakes below the EARs for all vitamins and minerals analysed (exception: vitamin B_9_).

### 3.3. Multivariate Analyses

After adjusting for the covariates, those with poor mental health status had an increased odds of being food insecure (OR = 1.60, 95% CI 1.45 to 1.71), having poor diet quality as measured by the HEI (1.61, 95% CI 1.34 to 1.81), protein intakes below the AMDR (OR = 1.01, 95% CI 1.00 to 1.02), and potentially inadequate intakes of folate (OR = 1.58, 95% CI 1.17 to 1.90) and iron (OR = 1.45, 95% CI 1.23 to 1.88) (Table 2).

## 4. Discussion

This study provided evidence that dietary quality was lower for those who were food insecure and had poor mental health. Based on stratified analysis by age and gender, significant associations were found between mental health and food insecurity for protein, fat, carbohydrates, fibre, vitamins A, B_1_, B_2_, B_3_, B_6_, B_9_, B_12_, C, and D, as well as the minerals magnesium, phosphorus, potassium, and zinc—particularly for older females. Furthermore, for those who were food insecure and had poor mental health, there was a higher prevalence of potentially inadequate intakes of most vitamins and minerals. When food insecurity, diet quality, and potentially inadequate intakes were simultaneously analysed with adjustment for covariates, food insecurity, poor diet quality, and suboptimal intakes of protein (marginal significance), folate, and iron all independently predicted poor mental health. Food insecurity and poor diet quality appeared to be the most significant predictors of poor mental health.

This study is the first to concurrently analyse food insecurity, diet quality, suboptimal nutrient intakes, and perceived mental health, and found results that are consistent with previous investigations that individually showed lower nutrient intakes, poor diet quality, and food insecurity are associated with poor perceived mental health [3,17,48,49]. In addition, our findings indicated that food insecurity is associated with dietary compromises that are of sufficient magnitude to impact nutritional and mental health, regardless of whether respondents did or did not have a diagnosed mental health condition.

Food insecurity tended to systematically drive increases in the proportion of individuals consuming a poor-quality diet and low intake levels of essential nutrients. Vitamins and minerals have important roles in various brain physiological processes, and have been linked with mental health [17]. Insufficient micronutrient levels can have harmful effects on the brain by reducing proteins such as brain-derived neurotropic factor (BDNF) and by up-regulating the stress response, immune, and oxidative systems [50]. Iron is involved in myelin production [51] and is a cofactor for neurotransmitter synthesis [52]. Zinc is involved in neuron migration, synaptogenesis, and neurogenesis [53]. Vitamins B_6_, B_9_, and B_12_ affect methylation in the central nervous system [54], and maintain the integrity of the myelin sheath [55]. Indeed, all dietary vitamins and minerals have critical roles in brain metabolism [15].

Multiple integrated mechanisms could explain the various findings reported here. For example, diet quality may represent the concerted action of various compounds within foods working synergistically [56] that support mental health. Individually, inadequate intakes of nutrients such as folate and iron independently predicted mental health, suggesting that there is a heightened need for some specific nutrients in relation to mental well-being. The results are particularly surprising for folate intakes, given that in 1998 the Government of Canada instituted mandatory folic acid fortification for all white flour and enriched pasta and cornmeal products [57], and the proportion of respondents with poor mental health consuming levels below the EAR did not differ significantly by food security status. While food insecurity encompasses components of dietary quality, many individual (e.g., stress and anxiety associated with attempts to access food), household (e.g., deprivation, parent’s placement of a child’s nutrition needs above theirs), and structural (e.g., poverty, stigma) drivers also explain independent links between food insecurity and mental health [58,59]. Clearly, future research is needed to help disentangle the complex relationships between nutritional and population mental health, which can then better direct food and health policies.

There are several limitations of this study. With the cross-sectional design, causality cannot be inferred. However, results from longitudinal studies suggest that bidirectional relationships occur between poor mental health (defined as depression), food insecurity, and diet quality [6,60]. The HFSSM focuses on income and does not consider factors such as diet self-efficacy (i.e., the belief in one’s ability to manage diet even in the face of obstacles such as stress) and other important psychosocial variables that relate to dietary behaviours [61,62]. The HFSSM also does not account for intra-household variations [63], and the measures of food insecurity (previous year) and diet quality (previous 24 h) reflected incongruent time frames. The reporting of suboptimal macronutrient, vitamin, and mineral nutrient intakes were confined to those where reference levels (e.g., EARs) exist and sufficient data was available to analyse. As is common with dietary surveys, underreporting did occur and may have overestimated prevalences of suboptimal intakes. However, no significant differences were observed in EI:EER and food security status among the age/gender categories. It is difficult to determine how these results may translate to other countries, as food insecurity and mental health measures vary. In national surveys, the US and Canada use versions of the HFSSM, and estimates of food insecurity in the US appear to be double that observed in Canada [64,65]. However, in Canada, food insecurity appears to have more of an impact on nutritional inadequacy [66]. None of the other industrialized countries use the same self-report mental health measure as the CCHS. The monitoring of self-reported mental health has only emerged in recent years, and this measure is increasingly recognized to be relevant as poor mental health has been associated with physical health, health service utilization, and psychiatric morbidities [67].

The results of this study suggest that alternatives for food and social policy need to be explored. In the Canadian context, there are limited publicly funded food programs that provide assistance to vulnerable households. In the US, for example, food subsidy programs such as the US Department of Agriculture’s Supplemental Nutrition Assistance Program (SNAP) for Women, Infants, and Children (WIC) exist to improve food security for low-income individuals and households. Evidence suggests that participation in these programs contributes to small increases in the intake of targeted nutrients and foods, and that the nutrition education component of the programs (SNAP-Ed) improves skills in food resource management [68,69,70]. Thus, contextual factors such as food and nutrition assistance programs, food supply, food pricing, and other policies and programs need to be explored as means to ameliorate food insecurity, improve diet quality, and foster mental health within populations.

## 5. Conclusions

Overall, the results suggested that food insecurity, poor diet quality, and inadequate intakes of selected micronutrients were independently associated with poor mental health in a general national sample. These findings suggest that public policies that support both food security and high quality dietary intakes could promote mental health and well-being. Population-based interventions that have the potential to improve diet quality and food security—such as the July 2016 Canadian Universal Child Care Benefit for families below specified income levels—should be evaluated in relation to mental health.

## Figures and Tables

**Table 1:** Prevalence estimates for categories of the acceptable macronutrient distribution ranges (AMDRs) and estimated average requirements (EARs) by mental health and food security ^a^.

Variable	Good Mental Health		Poor Mental Health	
	Food Secure	Food Insecure	Food Secure	Food Insecure
AMDRs				
Protein				
<20%	9.9	15.4 **	10.6	18.6 **
20% to 35%	52.7	51.7	55.4	53.1
>35%	37.4	32.9 **	34.0	28.3 **
Fat				
<45%	20.3	16.3 **	19.0	21.3
45% to 65%	52.8	58.4 **	56.9	64.5 **
>65%	26.4	25.2	24.1	14.2 **
EARs				
Vitamin A	52.3	62.8 **	54.4	67.0 **
Vitamin B_1_ (Thiamin)	18.1	21.7 **	24.4	39.2 **
Vitamin B_2_ (Riboflavin)	15.8	21.2 **	19.7	37.7 **
Vitamin B_3_ (Niacin)	2.5	4.3 **	6.4	8.7 **
Vitamin B_6_ (Pyridoxine)	26.7	33.0 **	39.6	42.5 **
Vitamin B_9_ (Folate)	79.4	82.7 **	87.1	87.2
Vitamin C	35.9	46.0 **	43.2	55.7 **
Iron	7.3	11.3 **	12.5	18.9 **
Magnesium	49.8	59.5 **	58.5	61.2 **
Phosphorus	10.4	17.1 **	16.7	23.7 **
Zinc	33.5	43.9 **	37.9	45.6 **

^a^ Carbohydrates and some micronutrients with EARs are not reported because stratified analysis created cell sizes < 5; ** *p* < 0.001; test-statistics (*z*-statistic) range from 2.56 to 75.75; *p*-values 0.011 to <0.0001.

**Table 2:** Logistic regression estimates of food insecurity, healthy eating index (HEI), acceptable macronutrient distribution ranges (AMDRs), and estimated average requirements (EARs) in relation to poor mental health.

Variable	Odds Ratio (95% CI)	*p*-Value
Food insecurity	1.60 (1.45–1.71)	0.0048
HEI		
Poor vs. good	1.61 (1.34–1.81)	0.0296
Needs improvement vs. good	1.06 (1.00–1.11)	0.2298
AMDRs		
Fat	1.00 (0.97–1.03)	0.8252
Protein	1.01 (1.00–1.02)	0.0312
EARs		
Vitamin A	0.88 (0.59–1.31)	0.5253
Vitamin B_1_ (Thiamin)	1.45 (0.98–1.13)	0.0604
Vitamin B_2_ (Riboflavin)	1.35 (0.66–2.76)	0.4056
Vitamin B_3_ (Niacin)	0.53 (0.21–1.32)	0.1727
Vitamin B_6_ (Pyridoxine)	0.79 (0.46–1.38)	0.4133
Vitamin B_9_ (Folate)	1.58 (1.17–1.90)	0.0148
Vitamin C	1.37 (0.90–2.08)	0.1376
Iron	1.45 (1.23–1.88)	0.0192
Zinc	1.35 (0.90–2.01)	0.1479

Significant associations were also indicated for income, smoking status, and marital status (*p* < 0.05).

## Supplementary Materials

The following are available online at http://www.mdpi.com/2072-6643/9/3/274/s1, Table S1: Intakes of energy and macronutrients from first 24-h dietary recall according to mental health and food security status; Table S2: Intakes of vitamins and minerals from first 24-h dietary recall according to mental health and food security status.
