# Supplementary material for: Food Insecurity, Poor Diet Quality, and Suboptimal Intakes of Folate and Iron Are Independently Associated with Perceived Mental Health in Canadian Adults
Source: Nutrients. 2017 Mar 14;9(3):274. doi: 10.3390/nu9030274 (PMC5372937; doi:10.3390/nu9030274)
Supplement: Supplementary file 1 [file nutrients-09-00274-s001.doc]

**Table S1.** Intakes of energy and macronutrients from first 24-hour dietary recall according to mental health (MH) and food security status.

| **Variable** | **Median (25^th^, 75^th^ percentile)** | | | | | | | | | | | |
| --- | --- | --- | --- | --- | --- | --- | --- | --- | --- | --- | --- | --- |
|  | **19–30 years** | | | | **31–50 years** | | | | **51–70 years** | | | |
|  | **Males** | | **Females** | | **Males** | | **Females** | | **Males** | | **Females** | |
|  | **Good MH** | **Poor MH** | **Good  MH** | **Poor MH** | **Good  MH** | **Poor MH** | **Good  MH** | **Poor MH** | **Good  MH** | **Poor MH** | **Good MH** | **Poor MH** |
| **Energy, kcal** | | | | | | | | | | | | |
| Food secure | 2556.1 (1876.4, 3368.2) | 2781.3 (1942.7, 3745.0) | 1781.3 (1331.0, 2334.0) | 1867.1 (1255.5, 2738.8) | 2326.7 (1730.1, 3089.3) | 2206.2 (1579.3, 2903.0) | 1677.8 (1233.1, 2224.0) | 1749.4 (1268.0, 2330.9) | 2074.4 (1560.2, 2690.0) | 2054.6 (1421.8, 2547.7) | 1566.8 (1183.4, 2021.2) | 1514.5 (1038.8, 1958.5) |
| Food insecure | 2342.5 (1734.0, 3205.7) | 2453.3 (1560.9, 3873.1) | 1741.0 (1250.1, 2347.5) | 1725.4 (1156.7, 2248.6) | 2219.7 (1521.8, 3150.6) | 1892.7 (1180.7, 2613.4) | 1590.7 (1028.7, 2106.8) | 1650.0 (1105.6, 2285.7) | 1761.2 (1184.0, 2444.7) | 1732.9 (1297.2, 2370.4) | 1243.9 (848.5, 1750.2) | 1444.6 (998.0, 1856.0) |
| **Energy Intake:Estimated Energy Requirements (EI:EER)** | | | | | | | | | | | | |
| Food secure | 0.81 (0.75, 0.84) | 0.79 (0.73, 0.82) | 0.80 (0.76, 0.83) | 0.78 (0.68, 0.81) | 0.82 (0.76, 0.85) | 0.80 (0.77, 0.84) | 0.78 (0.72, 0.83) | 0.75 (0.71, 0.77) | 0.79 (0.75, 0.83) | 0.77 (0.74, 0.82) | 0.82 (0.79, 0.86) | 0.79 (0.76, 0.82) |
| Food insecure | 0.72 (0.66, 0.78) | 0.68 (0.63, 0.74) | 0.72 (0.65, 0.78) | 0.67 (0.62, 0.73) | 0.72 (0.68, 0.77) | 0.70 (0.67, 0.74) | 0.77 (0.73, 0.80) | 0.75 (0.71, 0.78) | 0.76 (0.73, 0.79) | 0.75 (0.72, 0.78) | 0.75 (0.73, 0.77) | 0.71 (0.67, 0.74) |
| **Protein, g** | | | | | | | | | | | | |
| Food secure | 98.5 (70.3, 136.6) | 113.7 (53.6, 154.3) | 69.2 (47.9, 92.8) | 63.3 (39.8, 91.3) | 94.8 (66.3, 130.6) | 81.2 (58.3, 114.5) | 67.5 (48.3, 94.6) | 63.5 (34.0, 90.5) | 85.5 (62.5, 115.5) | 82.8 (56.7, 109.7) | 64.6 (47.1, 87.4) | 60.4 (38.6, 80.1) |
| Food insecure | 88.5 (60.5, 131.1) | 76.0 (50.7, 123.9) | 62.3 (39.2, 95.0) | 57.4 (37.2, 84.6) | 83.8 (49.6, 111.5) | 63.2 (27.9, 93.3)^*^ | 57.1 (38.2, 87.9) | 55.0 (37.9, 83.3) | 68.5 (43.1, 99.1) | 73.8 (42.7, 87.0)^*^ | 47.7 (33.2, 70.3) | 57.4 (33.6, 74.0) |
| **Protein, g/kg** | | | | | | | | | | | | |
| Food secure | 1.35 (1.23, 1.47) | 1.31 (1.20, 1.39) | 1.17 (1.11, 1.23) | 1.12 (1.06, 1.14) | 1.30 (1.22, 1.45) | 1.23 (1.14, 1.33) | 1.14 (1.07, 1.21) | 1.08 (0.99, 1.13) | 1.24 (1.14, 1.34) | 1.19 (1.11, 1.27) | 1.07 (1.01, 1.15) | 1.01 (0.98, 1.05) |
| Food insecure | 1.30 (1.21, 1.44) | 1.25 (1.16, 1.32) | 1.13 (1.07, 1.18) | 1.04 (0.99, 1.17) | 1.22 (1.13, 1.30) | 1.17 (1.09, 1.26) | 1.08 (1.03, 1.14) | 0.97 (0.93, 1.02) | 1.00 (0.96, 1.06) | 0.98 (0.94, 1.04) | 0.96 (0.88, 1.04) | 0.91 (0.85, 0.97) |

**Table S1.** Intakes of energy and macronutrients from first 24-hour dietary recall according to mental health and food security status /cont’d.

| **Variable** | **Median (25^th^, 75^th^ percentile)** | | | | | | | | | | | | | | | |
| --- | --- | --- | --- | --- | --- | --- | --- | --- | --- | --- | --- | --- | --- | --- | --- | --- |
|  | **19–30 years** | | | | **31–50 years** | | | | | | | | **51–70 years** | | | |
|  | **Males** | | **Females** | | **Males** | | | **Females** | | | | | **Males** | | **Females** | |
|  | **Good MH** | **Poor MH** | **Good  MH** | **Poor MH** | **Good  MH** | **Poor MH** | | **Good  MH** | | | **Poor**  **MH** | | **Good  MH** | **Poor MH** | **Good MH** | **Poor MH** |
| **Protein, % of total calories** | | | | | | | | | | | | | | | | |
| Food secure | 15.3  (12.2, 19.0) | 15.9 (11.4, 19.3) | 14.9 (11.9, 18.7) | 13.6 (10.3, 16.5) | 15.7 (12.7, 20.1) | 15.1 (11.5, 19.0) | | 16.0 (12.6, 20.0) | | | 13.5 (10.2, 18.2) | | 16.3 (13.0, 20.4) | 16.1 (13.1, 20.2) | 16.3 (13.0, 20.2) | 15.8 (12.1, 20.1) |
| Food insecure | 13.8 (11.3, 18.1) | 13.7 (11.0, 17.0) | 13.6 (10.6, 17.2) | 13.7 (11.7, 17.2) | 14.3 (10.6, 18.7) | 13.4 (10.8, 15.7) | | 14.2 (11.0, 18.7) | | | 13.8 (10.0, 17.0) | | 15.7 (11.4, 19.6) | 14.0 (11.0, 16.0) | 15.2 (11.9, 19.6) | 15.9 (12.9, 18.8) |
| **Carbohydrates, g** | | | | | | | | | | | | | | | | |
| Food secure | 271.0 (164.1, 461.8) | 297.3 (142.1, 495.8) | 172.5 (98.8, 289.3) | 167.3 (90.7, 301.7) | 265.4 (154.5, 459.0) | | 236.9 (148.3, 518.9) | 180.0 (104.4, 327.4) | | | | 168.8 (76.3, 281.9) | 248.8 (147.5, 458.5) | 231.0 (126.4, 373.4) | 177.8 (103.9, 314.1) | 153.5 (91.5, 255.2) |
| Food insecure | 285.2 (116.8, 487.1) | 179.7 (90.9, 385.7) | 173.7 (89.1, 292.9) | 145.7 (56.0, 195.9) | 215.3 (121.7, 376.0) | | 229.4 (56.0, 405.6) | 172.0 (83.9, 306.5) | | | | 139.9 (75.3, 231.7)^*^ | 213.2 (80.2, 348.1) | 292.1 (121.8, 409.9) | 126.0 (60.9, 252.1) | 112.4 (93.3, 235.7)^*^ |
| **Carbohydrates, % of total calories** | | | | | | | | | | | | | | | | |
| Food secure | 49.0 (41.5, 56.3) | 48.4 (41.2, 53.8) | 52.0 (44.6, 59.1) | 53.3 (47.8, 60.0) | 46.8 (39.5, 54.6) | | 48.4 (40.4, 57.7) | 49.0 (41.3, 56.4) | | 50.8 (43.6, 57.7) | | | 46.3 (39.1, 53.8) | 49.2 (42.2, 55.4) | 49.6 (42.0, 56.8) | 50.6 (42.9, 59.1) |
| Food insecure | 49.4 (41.4, 57.6) | 47.0 (41.2, 55.1) | 50.6 (43.7, 58.7) | 51.4 (46.5, 60.4) | 49.2 (39.1, 56.7) | | 53.3 (43.7, 61.6) | 50.7 (44.1, 58.3) | | 52.8 (44.7, 59.3) | | | 47.8 (39.8, 56.8) | 52.5 (47.7, 61.8) | 52.6 (44.1, 60.8) | 53.3 (45.1, 59.0) |
| **Fat, g** | | | | | | | | | | | | | | | | |
| Food secure | 88.6 (58.4, 125.9) | 101.7 (66.6, 151.7) | 59.0 (40.6, 85.3) | 65.3 (42.2, 94.8) | 82.9 (55.1, 121.5) | | 71.4 (47.0, 111.0) | 59.2 (38.7, 86.1) | 61.9  (36.7, 84.7) | | | | 73.4 (48.6, 105.1) | 67.3 (37.2, 97.1) | 55.1 (36.4, 77.7) | 50.0 (31.2, 72.5) |
| Food insecure | 81.3 (52.4, 118.9) | 80.4 (43.3, 132.6) | 65.0 (39.6, 90.1) | 62.6 (33.5, 95.7) | 78.1 (49.5, 108.4) | | 66.3 (41.9, 100.6) | 55.9 (35.3, 82.1) | 53.9  (35.7, 85.7)^*^ | | | | 57.1 (40.6, 89.4) | 66.3 (44.2, 89.3) | 38.1 (24.5, 69.6) | 48.9 (25.3, 68.7)^*^ |

**Table S1.** Intakes of energy and macronutrients from first 24-hour dietary recall according to mental health and food security status /cont’d.

| **Variable** | **Median (25^th^, 75^th^ percentile)** | | | | | | | | | | | |
| --- | --- | --- | --- | --- | --- | --- | --- | --- | --- | --- | --- | --- |
|  | **19–30 years** | | | | **31–50 years** | | | | **51–70 years** | | | |
|  | **Males** | | **Females** | | **Males** | | **Females** | | **Males** | | **Females** | |
|  | **Good MH** | **Poor MH** | **Good  MH** | **Poor MH** | **Good  MH** | **Poor MH** | **Good  MH** | **Poor MH** | **Good  MH** | **Poor MH** | **Good MH** | **Poor MH** |
| **Fat, % of total calories** | | | | | | | | | | | | |
| Food secure | 30.9 (24.8, 36.8) | 34.1 (26.5, 39.9) | 30.1 (24.0, 36.5) | 31.8 (24.9, 36.3) | 32.4 (25.9, 38.9) | 32.7 (23.9, 38.6) | 31.8 (25.3, 38.4) | 31.7 (24.3, 38.4) | 32.5 (25.6, 38.5) | 30.6 (22.6, 36.5) | 31.4 (25.3, 37.8) | 31.7 (24.3, 38.4) |
| Food insecure | 31.7 (25.6, 37.7) | 29.8 (24.5, 36.3) | 33.1 (25.6, 39.6) | 33.5 (23.9, 38.6) | 31.6 (25.9, 39.1) | 31.8 (22.5, 38.8) | 32.0 (25.7, 38.0) | 29.3 (25.6, 37.9) | 31.9 (21.3, 38.6) | 30.5 (23.1, 36.2) | 29.9 (23.3, 38.1) | 29.5 (36.0, 22.8) |
| **Fiber, g** | | | | | | | | | | | | |
| Food secure | 16.0 (10.7, 23.4) | 18.0 (10.9, 28.6) | 12.9  (8.7,  18.8) | 12.7 (7.9, 19.9) | 16.3 (11.2, 23.6) | 14.5 (8.9, 23.4) | 13.7  (9.0,  19.7) | 12.6 (7.8, 18.0) | 16.6 (11.2, 23.9) | 14.0 (10.1, 21.3) | 14.4 (9.7, 20.8) | 12.4 (7.7, 17.0) |
| Food insecure | 14.8 (9.5, 20.6) | 11.8 (10.1, 20.6) | 10.8  (6.7,  16.6) | 12.5 (7.3, 16.3) | 14.8  (9.2,  20.3) | 11.1 (7.5, 17.1)^*^ | 11.9  (7.7,  17.5) | 11.9 (6.5, 17.0) | 11.9  (7.6,  19.0) | 13.2 (9.3, 16.7) | 10.4 (6.8, 16.8) | 10.1 (6.2, 17.2)^*^ |

^*^*p* < 0.05 among the age/gender categories of mental health and food security status by gender based on Kruskal–Wallis tests (test statistics for significant differences ranged from 2.00 to 4.50; *p*-values 0.045 to 0.0002)

**Table S2.** Intakes of vitamins and minerals from first 24-hour dietary recall according to mental health and food security status.

| **Variable** | **Median (25^th^, 75^th^ percentile)** | | | | | | | | | | | |
| --- | --- | --- | --- | --- | --- | --- | --- | --- | --- | --- | --- | --- |
|  | **19–30 years** | | | | **31–50 years** | | | | **51–70 years** | | | |
|  | **Males** | | **Females** | | **Males** | | **Females** | | **Males** | | **Females** | |
|  | **Good MH** | **Poor MH** | **Good MH** | **Poor MH** | **Good MH** | **Poor MH** | **Good MH** | **Poor MH** | **Good MH** | **Poor MH** | **Good MH** | **Poor MH** |
| **Vitamins** | | | | | | | | | | | | |
| **Vitamin A, RAE** | | | | | | | | | | | | |
| Food secure | 543.1  (294.1,  897.0) | 576.3  (311.4,  892.9) | 466.5  (265.0,  807.5) | 408.2  (207.1,  723.1) | 536.9  (300.4,  868.5) | 491.4  (261.6,  709.6) | 483.5  (271.2,  799.9) | 430.8  (230.4,  708.7) | 545.6  (321.4,  898.6) | 487.5  (194.3,  812.6) | 496.1  (299.3,  817.7) | 359.3  (198.8,  614.4) |
| Food insecure | 467.8  (223.1,  921.5) | 279.6  (178.0,  493.8) | 356.0  (168.2,  638.2) | 290.7  (157.7,  597.2) | 426.7  (223.4,  759.8) | 425.4  (139.0,  682.0) | 362.0  (214.9,  663.4) | 334.5  (181.7,  523.7) | 339.8  (161.3,  622.6) | 429.6  (263.5,  759.7) | 319.2  (192.8,  629.6) | 375.2  (209.8,  694.3) ^*^ |
| **Vitamin B_1_ (Thiamin), mg** | | | | | | | | | | | | |
| Food secure | 1.9  (1.3, 2.7) | 2.1  (1.4, 2.9) | 1.4  (1.0, 2.0) | 1.3  (0.9, 1.9) | 1.8  (1.2, 2.5) | 1.6  (1.1, 2.5) | 1.3  (0.9, 1.8) | 1.1  (0.6, 1.6) | 1.7  (1.2, 2.3) | 1.6  (1.2, 2.1) | 1.3  (0.9, 1.8) | 1.2  (0.7, 1.7) |
| Food insecure | 1.7  (1.1, 2.5) | 1.9  (1.1, 2.1) | 1.2  (0.8, 1.8) | 1.1  (0.8, 1.7) | 1.6  (1.0, 2.2) | 1.3  (0.9, 2.1)^*^ | 1.1  (0.8, 1.7) | 1.2  (0.9, 1.6)^*^ | 1.4  (0.9, 2.2) | 1.4  (1.0, 2.2) | 1.0  (0.7, 1.5) | 1.1  (0.8, 1.6)^*^ |
| **Vitamin B_2_ (Riboflavin), mg** | | | | | | | | | | | | |
| Food secure | 2.1  (1.5, 3.0) | 2.0  (1.4, 3.2) | 1.6  (1.1, 2.2) | 1.4  (1.1, 2.1) | 2.0  (1.4, 2.7) | 1.9  (1.3, 2.7) | 1.5  (1.1, 2.1) | 1.5  (1.0, 2.0) | 1.9  (1.4, 2.5) | 1.8  (1.3, 2.5) | 1.5  (1.1, 2.0) | 1.3  (0.9, 1.9) |
| Food insecure | 2.0  (1.2, 2.7) | 2.0  (1.3, 2.8) | 1.4  (0.9, 2.0) | 1.4  (0.8, 1.9) | 1.7  (1.2, 2.5) | 1.6  (0.9, 2.2) | 1.4  (0.9, 1.9) | 1.4  (1.0, 1.8) | 1.6  (0.9, 2.1) | 1.5  (1.1, 1.9) | 1.1  (0.8, 1.7) | 1.2  (0.9, 1.9)^*^ |
| **Vitamin B_3_** (**Niacin), mg** | | | | | | | | | | | | |
| Food secure | 45.2  (33.2, 61.4) | 51.4  (28.7, 65.5) | 31.5  (22.8,  41.8) | 29.2  (20.1,  43.2) | 44.0  (32.0,  60.4) | 37.7  (26.7,  55.4) | 31.7  (22.6,  43.2) | 28.3  (18.3.  39.9) | 40.0  (29.6,  54.2) | 38.4  (29.3,  51.4) | 30.0  (22.1,  40.1) | 27.5  (20.2,  36.1) |
| Food insecure | 42.0  (29.9,  57.0) | 38.3  (24.0,  62.7) | 29.3  (20.1,  39.9) | 26.0  (17.8,  38.2) | 41.8  (26.6,  55.4) | 28.9  (15.2,  42.8)^*^ | 28.0  (18.9,  40.6) | 27.3  (18.7,  39.1) | 34.7  (23.4,  46.5) | 31.7  (21.2,  42.9) | 22.4  (16.2,  34.5) | 26.3  (19.0,  37.3)^*^ |
| **Vitamin B_6_ (Pyridoxine), mg** | | | | | | | | | | | | |
| Food secure | 2.0  (1.4, 2.9) | 2.0  (1.0, 2.9) | 1.5  (1.0, 2.1) | 1.2  (0.8, 1.8) | 2.0  (1.4, 2.8) | 1.8  (1.2, 2.8) | 1.5  (1.0, 2.1) | 1.4  (0.8, 1.9) | 1.9  (1.3, 2.7) | 1.9  (1.2, 2.5) | 1.5  (1.0, 2.1) | 1.3  (0.8, 1.9) |
| Food insecure | 1.7  (1.1, 2.3) | 1.7  (1.2, 2.4) | 1.2  (0.8, 1.8) | 0.9  (0.6, 1.6) | 1.7  (1.1, 2.5) | 1.1  (0.5, 2.0)^*^ | 1.3  (0.7, 2.0) | 1.3  (0.7, 1.8) | 1.5  (0.9, 2.4) | 1.6  (0.9, 2.0) | 1.1  (0.6, 1.7) | 1.0  (0.7, 1.5)^*^ |

**Table S2.** Intakes of vitamins and minerals from first 24-hour dietary recall according to mental health and food security status /cont’d.

| **Variable** | **Median (25^th^, 75^th^ percentile)** | | | | | | | | | | | |
| --- | --- | --- | --- | --- | --- | --- | --- | --- | --- | --- | --- | --- |
|  | **19–30 years** | | | | **31–50 years** | | | | **51–70 years** | | | |
|  | **Males** | | **Females** | | **Males** | | **Females** | | **Males** | | **Females** | |
|  | **Good MH** | **Poor MH** | **Good MH** | **Poor MH** | **Good MH** | **Poor MH** | **Good MH** | **Poor MH** | **Good MH** | **Poor MH** | **Good MH** | **Poor MH** |
| **Vitamins** | | | | | | | | | | | | |
| **Vitamin B_9_ (Folate), µg** | | | | | | | | | | | | |
| Food secure | 334.2  (501.4,  718.4) | 359.1  (586.4,  910.3) | 240.2  (362.7,  526.6) | 245.5  (419.6,  556.2) | 316.0  (458.9,  658.2) | 280.5  (403.7,  572.5) | 244.8  (356.9,  514.2) | 220.1  (334.3,  503.8) | 300.9  (425.1,  582.5) | 275.8  (390.3,  504.7) | 249.7  (351.7,  484.9) | 204.3  (291.0,  421.5) |
| Food insecure | 329.1  (454.6,  643.0) | 237.7  (572.1,  689.7) | 200.7  (321.3,  488.2) | 192.8  (300.3,  521.5) | 277.0  (452.4,  630.0) | 233.8  (380.1,  584.0)^*^ | 211.5  (327.1,  461.5) | 206.3  (316.2,  450.1)^*^ | 221.3  (379.0,  532.8) | 287.0  (336.0,  472.2) | 173.1  (264.3,  381.9) | 190.7  (291.5,  417.7)^*^ |
| **Vitamin B_12_ (Cyanocobalamin), µg** | | | | | | | | | | | | |
| Food secure | 4.1  (2.4, 6.8) | 3.6  (1.8, 7.0) | 2.7  (1.5, 4.4) | 2.3  (1.3, 3.7) | 3.8  (2.2, 6.5) | 3.5  (2.0, 6.2) | 2.6  (1.5, 4.4) | 2.4  (1.2, 4.0) | 3.6  (2.1, 6.2) | 3.6  (2.0, 5.2) | 2.7  (1.5, 4.3) | 2.1  (1.1, 3.7) |
| Food insecure | 3.4  (1.6, 6.4) | 2.2  (1.3, 5.3) | 2.3  (1.1, 4.2) | 2.3  (1.2, 3.6) | 3.1  (1.5, 6.2) | 2.0  (1.1, 4.1) | 2.2  (1.2, 3.8) | 2.5  (1.0, 4.0) | 2.4  (1.2, 4.7) | 2.2  (1.4, 5.0) | 1.8  (1.1, 3.1) | 2.1  (1.2, 4.2)^*^ |
| **Vitamin C, mg** | | | | | | | | | | | | |
| Food secure | 90.7  (38.3,  206.0) | 72.6  (27.5,  156.0) | 96.0  (38.2,  189.7) | 72.8  (31.9,  133.8) | 90.4  (39.5,  182.6) | 82.5  (27.7,  142.0) | 84.2  (35.4,  158.5) | 68.6  (31.0,  146.3) | 88.1  (39.2,  163.5) | 66.5  (27.2,  124.5) | 91.8  (42.8,  157.4) | 65.9  (26.2,  145.0) |
| Food insecure | 65.0  (25.4,  164.5) | 58.4  (27.7,  245.3) | 68.0  (28.0,  147.8) | 48.8  (14.4,  112.9)^*^ | 52.5  (26.2,  118.9) | 46.3  (9.3,  132.4) | 51.3  (20.4,  128.2) | 44.5  (24.6,  91.7) | 42.6  (19.4,  108.6) | 81.1  (26.3,  111.9) ^*^ | 55.4  (19.5,  122.1) | 40.8  (17.1,  104.0)^*^ |
| **Vitamin D, µg** | | | | | | | | | | | | |
| Food secure | 4.8  (2.2, 8.4) | 6.0  (2.6, 9.1) | 3.5  (1.7, 6.3) | 2.9  (1.5, 5.5) | 4.4  (2.2, 7.6) | 4.1  (2.3, 8.1) | 3.6  (1.7, 6.3) | 2.8  (1.6, 5.4) | 4.6  (2.6, 7.6) | 4.2  (2.4, 7.2) | 3.7  (2.1, 6.1) | 3.1  (1.3, 5.3) |
| Food insecure | 4.3  (2.2, 8.1) | 2.2  (1.4, 5.3) | 3.0  (1.3, 5.8) | 3.0  (1.6, 6.7) | 3.9  (2.1, 6.6) | 3.6  (1.6, 7.1) | 3.1  (1.4, 5.9) | 3.4  (1.4, 6.1) | 3.3  (1.7, 6.6) | 3.3  (1.6, 6.5) | 3.3  (1.5, 5.2) | 3.1  (1.7, 5.6)^*^ |

**Table S2.** Intakes of vitamins and minerals from first 24-hour dietary recall according to mental health and food security status /cont’d.

| **Variable** | **Median (25^th^, 75^th^ percentile)** | | | | | | | | | | | |
| --- | --- | --- | --- | --- | --- | --- | --- | --- | --- | --- | --- | --- |
|  | **19–30 years** | | | | **31–50 years** | | | | **51–70 years** | | | |
|  | **Males** | | **Females** | | **Males** | | **Females** | | **Males** | | **Females** | |
|  | **Good MH** | **Poor MH** | **Good MH** | **Poor MH** | **Good MH** | **Poor MH** | **Good MH** | **Poor MH** | **Good MH** | **Poor MH** | **Good MH** | **Poor MH** |
| **Minerals** | | | | | | | | | | | | |
| **Calcium, mg** | | | | | | | | | | | | |
| Food secure | 941.8  (573.3,  1465.6) | 1022.0  (618.8.  1611.3) | 767.6  (455.6,  1177.9) | 605.3  (422.1,  1194.0) | 808.5  (497.6,  1217.2) | 726.3  (431.7,  1126.1) | 687.4  (422.3.  1057.0) | 628.6  (376.0,  965.9) | 712.9  (464.2,  1068.5) | 683.1  (421.5,  988.9) | 647.3  (418.3,  959.5) | 575.5  (308.0,  851.8) |
| Food insecure | 802.0  (429.6,  1290.4) | 718.9  (519.2,  1210.2) | 603.2  (325.3,  1082.0) | 562.9  (351.0,  1028.1) | 641.9  (407.8,  905.1) | 568.7  (349.7,  905.5) | 578.6  (343.1,  896.2) | 567.7  (394.8,  951.4) | 521.5  (270.1,  812.1) | 504.0  (323.7,  857.2) | 457.1  (263.3,  735.0) | 527.9  (329.0,  792.5) |
| **Iron, mg** | | | | | | | | | | | | |
| Food secure | 17.3 (15.6, 19.2) | 17.1 (15.4, 19.0) | 13.6 (12.3, 15.2) | 12.9 (11.8, 14.7) | 16.8 (14.7, 17.6) | 16.1 (14.5, 17.9) | 13.3 (12.1, 14.8) | 12.7 (11.7, 14.3) | 15.3 (13.6, 16.2) | 14.7 (12.9, 15.4) | 12.4 (11.5, 13.9) | 11.8  (10.9,  12.7) |
| Food insecure | 16.7 (15.3, 18.9) | 16.2 (15.0, 18.4) | 11.1 (10.3, 12.1) | 10.7 (10.0, 11.8) | 15.1 (14.8, 16.2) | 14.6 (13.7, 15.8) | 11.3 (10.6, 12.4) | 10.8  (9.9,  11.6) | 12.4 (11.5, 13.4) | 11.7 (11.1, 12.3) | 11.0 (10.1, 11.9) | 10.5  (9.8,  11.6) |
| **Magnesium, mg** | | | | | | | | | | | | |
| Food secure | 240.0  (337.6,  463.5) | 248.0  (343.6,  544.2) | 194.3  (263.5,  357.0) | 181.4  (265.9,  334.5) | 252.6  (335.7,  450.4) | 232.6  (323.2,  453.5) | 274.9  (199.4,  371.8) | 254.0  (189.4,  364.4) | 321.9  (241.1,  429.7) | 309.7  (222.3,  391.4) | 271.2  (200.5,  364.9) | 248.3  (173.3,  332.6) |
| Food insecure | 299.5  (203.4,  430.6) | 336.7  (219.4,  440.4) | 220.7  (152.5,  302.8) | 241.3  (157.7,  273.9) | 298.8  (217.6,  410.7) | 214.7  (166.4,  338.6) | 246.4  (180.3,  331.5) | 244.2  (157.3,  331.9) | 264.3  (182.8,  336.9) | 244.2  (156.9,  387.8) | 215.9  (143.2,  299.3) | 227.9  (150.7,  320.2)^*^ |
| **Phosphorus, mg** | | | | | | | | | | | | |
| Food secure | 1539.5  (1082.0,  2141.6) | 1592.0  (939.4,  2409.9) | 1121.6  (815.3,  1551.2) | 1023.6  (712.8,  1577.4) | 1428.2  (1012.0,  1951.0) | 1303.2  (939.9,  1973.1) | 1104.5  (778.2,  1519.0) | 1047.0  (688.4,  1435.0) | 1306.6  (954.9,  1769.7) | 1182.2  (904.5,  1758.9) | 1049.4  (766.9,  1431.9) | 899.8  (625.7,  1283.3) |
| Food insecure | 1325.8  (903.3,  1859.4) | 1206.3  (818.9,  1598.2) | 1000.8  (634.0,  1438.8) | 1038.3  (679.9,  1316.7) | 1221.2  (826.3,  1680.2) | 1032.5  (600.2,  1683.3)^*^ | 928.7  (596.8,  1377.2) | 968.9  (629.5,  1303.9) | 997.6  (724.4,  1392.9) | 916.6  (688.0,  1681.1) | 798.3  (499.1,  1210.7) | 896.1  (613.5,  1264.7)^*^ |

**Table S2.** Intakes of vitamins and minerals from first 24-hour dietary recall according to mental health and food security status /cont’d.

| **Variable** | **Median (25^th^, 75^th^ percentile)** | | | | | | | | | | | |
| --- | --- | --- | --- | --- | --- | --- | --- | --- | --- | --- | --- | --- |
|  | **19–30 years** | | | | **31–50 years** | | | | **51–70 years** | | | |
|  | **Males** | | **Females** | | **Males** | | **Females** | | **Males** | | **Females** | |
|  | **Good MH** | **Poor MH** | **Good MH** | **Poor MH** | **Good MH** | **Poor MH** | **Good MH** | **Poor MH** | **Good MH** | **Poor MH** | **Good MH** | **Poor MH** |
| **Minerals** | | | | | | | | | | | | |
| **Potassium, mg** | | | | | | | | | | | | |
| Food secure | 3219.5  (2314.1,  4552.8) | 2848.2  (1877.0,  4529.2) | 2556.5  (1843.2,  3508.1) | 2426.0  (1720.2,  3249.8) | 3314.2  (2423.7,  4444.6) | 3052.6  (2273.9,  4377.5) | 2670.5  (1911.7,  3563.8) | 2625.5  (1843.4,  3426.0) | 3226.8  (2359.1,  4218.4) | 2131.2  (3069.1,  3777.1) | 1977.8  (2674.2,  3503.2) | 2360.3  (1569.5,  3175.8) |
| Food insecure | 2911.1  (2053.4,  3953.9) | 3182.8  (1397.4,  4426.3) | 2196.6  (1563.0,  3184.7) | 1928.6  (1428.7,  2725.1) | 3036.4  (2004.1,  4127.1) | 2387.3  (1259.2,  3760.6)^*^ | 2315.2  (1596.3,  3284.8) | 2136.6  (1454.3,  3292.4) | 2374.2  (1825.1,  3446.8) | 2462.6  (1658.8,  3567.8) | 2132.9  (1469.4,  2938.8) | 2278.6  (1455.4,  2746.2)^*^ |
| **Sodium, mg** | | | | | | | | | | | | |
| Food secure | 2573.4  (3740.8,  5131.6) | 2426.1  (3844.1,  5340.7) | 1769.5  (2577.9,  3543.0) | 1701.1  (2678.0,  3898.6) | 2280.1  (3406.6,  4765.0) | 2075.3  (3284.1,  4546.7) | 1675.6  (2469.0,  3506.9) | 1489.7  (2363.7,  3326.2) | 2165.9  (3038.5,  4201.3) | 2125.0  (2919.1,  3932.9) | 1654.5  (2317.6,  3162.5) | 1340.5  (2089.0,  3177.2) |
| Food insecure | 3445.9 (2307.5,  5008.7) | 2932.9  (1914.4,  5274.5) | 2495.0  (1656.9,  3687.7) | 2544.9  (1476.6,  3805.3) | 3132.5  (2197.3,  4222.4) | 3038.7  (1598.7,  4242.5) | 2287.0  (1530.7,  3391.3) | 2566.3  (1706.4,  3468.5) | 2363.9  (1624.1,  3672.5) | 3461.3  (1335.9,  4250.1) | 1985.4  (1252.5,  2849.7) | 2295.8  (1435.8,  3048.5) |
| **Zinc, mg** | | | | | | | | | | | | |
| Food secure | 12.6  (8.6,  18.1) | 13.6  (7.5,  20.8) | 8.7  (6.1,  12.2) | 9.1  (5.3,  12.4) | 12.2  (8.2,  17.3) | 11.5  (7.2,  17.1) | 8.6  (6.0,  12.3) | 8.4  (5.2,  12.2) | 10.9  (7.6,  15.3) | 10.7  (6.9,  14.7) | 8.3  (5.9,  11.6) | 7.8  (4.6,  11.2) |
| Food insecure | 12.0  (7.6,  18.1) | 9.9  (6.3,  19.2) | 8.1  (5.3,  11.9) | 7.4  (5.0,  11.3) | 10.4  (6.2,  15.8) | 8.8  (4.2, 11.8)^*^ | 7.4  (5.0,  11.4) | 7.8  (4.6,  11.3) | 7.9  (5.5,  13.6) | 8.2  (4.5,  12.9) | 6.3  (4.5,  9.6) | 6.8  (4.8,  10.5) |

^*^*p* < 0.05 between mental health and food security status within the designated gender group based on Kruskal–Wallis tests (test statistics for significant differences ranged from 2.07 to 4.50; *p*-values 0.038 to 0.00007)
